# Strategies used by nurses and tracheostomized users in communication: systematic review

**DOI:** 10.1080/07853890.2021.1896071

**Published:** 2021-09-28

**Authors:** Ana Rita Verissimo Patrão Duarte, Cidália Maria da Cruz Silva Patacas de Castro, Inês dos Mártires Nobre, Joana Mafalda dos Santos Cordeiro, Mafalda Luís Pereira da Costa

**Affiliations:** a Nurse Department, Escola Superior de Saúde Egas Moniz (ESSEM), Egas Moniz Cooperativa de Ensino Superior, Caparica, Portugal

## Abstract

Communication is a basic human need necessary to establish a relationship of trust between patients and multidisciplinary team, especially with nursing team. With this, it is necessary to find strategies that allows not only the tracheostomized patients but also nurses to communicate in an effective way [1].

This is a systematic review that aims to give an answer to our investigation question: “Which strategies are used by nurses and tracheostomized patients in communication?” To understand which strategies are used by nurses and tracheostomized patients in communication. Our research was based on electronic data such as B-On, SciELO, RCAAP *and EBSCOhost*. According to our descriptors: communication, strategies, nursing and tracheostomy, we included articles published between 2011 and 2019, in Portuguese or English. Seven articles were included in this systematic review.

From the analysis of the data emerged three fundamental themes regarding the strategies used by nurses and tracheostomized patients in communication, that are: (1) Communication strategies used; (2) Communication facilitators; (3) Difficulty in communication and social interaction. Regarding the first theme, we consider that the alternative communication strategies used by tracheostomized individuals involve gestures or writing, lip reading, illustrative cards, the speech valve and tracheostomy occlusion [2]. According to the second theme, we found that the user must be encouraged to speak clearly and slowly, taking into account key words or phrases (that may give clues) and to blink differently for yes or no [3]. Regarding the last theme, the difficulties shown by the tracheostomy user go through the inability to change the volume and tone of voice, thereby affecting social interaction with others [4]. The knowledge of health professionals regarding the stress factor of these users leads to a lack of expressiveness due to the inability to communicate. The communication of these two actors is considered a key element, helping to reassure the user [5]. For all these strategies, the positioning of the user and the receptor of the message is a primary factor [2]. The need for strategies to overcome the difficulties experienced by tracheostomized users provides a consistent basis for promotion quality nursing care [1,2].

The studies analysed indicate that currently, despite the existence of several communication techniques between the user and the other health professionals, (namely nurses), there is still some difficulty in teaching them. This difficulty has implications for the continuity of health care and affects not only users, but also their family and all professionals with whom they are in contact during hospitalisation. This review is important because it contributes to the improvement of nursing care and the compliance of the user.
